# Dimensions of physical activity as related to child attention-deficit/hyperactivity disorder symptoms and impairment

**DOI:** 10.1177/13591045211058338

**Published:** 2021-12-07

**Authors:** Katrina Aranas, Jacqueline P Leighton

**Affiliations:** 1 Educational Psychology, 3158 University of Alberta, Edmonton, Canada

**Keywords:** attention-deficit/hyperactivity disorder, physical activity, exercise, treatment, child psychopathology

## Abstract

In efforts to explore adjunct/alternative treatments for ADHD, this study investigated the associations between dimensions of physical activity (PA) and children’s ADHD symptoms and impairment. Current evidence-based treatments include medication and behaviour management, but there is widespread consensus that more treatment options are desirable. Although there is increasing support for PA as an adjunct/alternative to existing treatment for ADHD, the interplay of specific dimensions of PA has not been studied. Fifty-one parents of children aged 6–12 years with ADHD filled out questionnaires. Hierarchical regression analysis indicated that only some dimensions of PA explained a statistically significant portion of the variance in ADHD symptoms, beyond that explained by typical demographic variables. PA dimensions did not account for a statistically significant portion of ADHD impairment. Refining the measurement of how long children have engaged in PA is a key step in generating evidence for PA as an adjunct or alternate treatment for ADHD, and developing guidelines to manage parental expectations for this treatment in the benefit of their children.

Attention-deficit/hyperactivity disorder is the most common neurodevelopmental disorder in preschool-aged and school-aged children (Gordon Millichap, 2011) and the third most common mental health disorder, following depression and anxiety (Polanczyk et al., 2015). ADHD significantly impairs children’s attention and self-regulatory behaviours, often with concomitant social and emotional difficulties (Classi et al., 2012). According to the Centre for ADHD Awareness (https://caddac.ca/understanding-adhd/in-general/facts-stats-myths/), a conservative estimate of the worldwide prevalence rate of ADHD is 5%. ADHD is also negatively associated with health-related quality of life scores, diet and sports participation (Ahn et al., 2017) and positively associated with odds of screen time, obesity, anxiety and depression.

The American Academy of Pediatrics indicates the current established evidence-based treatments for alleviating symptoms of ADHD are medication and behaviour modification, preferably in combination (Subcommittee on Attention-Deficit/Hyperactivity Disorder, Steering Committee on Quality Improvement and Management, 2011). There is ample literature on how stimulant medications decrease inattention, hyperactivity, impulsivity and associated disruptive behaviours (Conners, 2002) and increase on-task behaviour, cooperation, predisposition and self-esteem (Smith & Shapiro, 2015). However, there are limitations with using stimulant medications. For example, stimulants have little effect on the academic achievement and peer relationship skills of children with ADHD; and about 30% do not show clinical improvements (Chronis et al., 2006). There are also issues of accessibility due to cost (Smith & Shapiro, 2015).

Behaviour modification is another treatment for ADHD, with some meeting the rigorous requirements for efficacy, effectiveness and clinical utility (Flay et al., 2005). Although behaviour modification therapy can be beneficial at home, in schools and across peer settings (Fabiano et al., 2009), it is costly and takes up significant time (Subcommittee on Attention-Deficit/Hyperactivity Disorder, Steering Committee on Quality Improvement and Management, 2011). Behavioural interventions also place a heavy burden on the adult in charge of implementations to maintain high levels of fidelity and intensity (Chronis et al., 2001). In fact, even behavioural parent training is associated with poor adherence to treatment on the part of families (Jones & Rabinovitch, 2014).

Challenges with current treatments have led to consideration of alternate treatments. For example, physical activity (PA) is defined as a contrived skeletal movement associated with an increase in the use of energy (Dishman et al., 2006; Rommel et al., 2015). PA involves, but is not limited to, leisure activities, spontaneous PA, organized PA classes, and sport competition, and has been proposed as a promising form of adjunct/alternative treatment for ADHD. Although PA might be considered highly similar to behaviour modification because both involve physical behaviour, PA involves increases to a child’s heart rate, breathing and consequent release of endorphins.

Three empirical arguments support the potential utility of PA in children with ADHD: (1) PA influences the same catecholaminergic systems as those targeted by stimulant medications (Wigal et al., 2012); (2) PA has been found to be correlated with reductions in anxiety and depression among children and improvement in socio-emotional functioning (Biddle & Asare, 2011; Korczak et al., 2017) and (3) PA is associated with improved executive functioning among typically developing children (Mavilidi et al., 2016; Verburgh et al., 2013). Indeed, PA has also been associated with the reduction of the following ADHD symptoms: inattention, hyperactivity and impulsivity (e.g. Cerrillo-Urbina et al., 2015; Jensen & Kenny, 2004; Suarez-Manzano & Ruiz-Ariza, 2018).

Although most of these studies are correlational, some employ experimental designs and show a reduction in ADHD symptoms from PA treatments (Hoza et al., 2015; Jensen & Kenny, 2004; Kang et al., 2011; Verret et al., 2012). The main PA variables manipulated are intensity, frequency and duration (Berwid & Halperin, 2012). Specifically, PA frequency has been manipulated to range from one to seven sessions per week; PA duration has been varied between 20 to 90 minutes per session; and PA length has been varied between 1 day to 12 weeks. However, little is known about the collective interplay of intensity, frequency and duration of PA and children’s ADHD symptoms and impairment (Cerrillo-Urbina et al., 2015; Cornelius et al., 2017; Reeves & Bailey, 2016; Verret et al., 2012).

The purpose of the current study is to examine the interplay between intensity, frequency and duration of physical activity (PA) and children’s ADHD symptoms and impairment, based on parent reports. Specifically, three hypotheses guided this study: (1) Parental reports of children’s PA levels account for children’s ADHD symptoms and/or impairment, (2) Parental reports of children’s engagement in PA account for children’s PA levels and (3) Higher reported-levels of PA and/or PA engagement by parents are associated with lower levels of children’s ADHD symptoms and/or impairments. Investigating how intensity, frequency and duration of PA relate to ADHD symptoms and/or impairment is necessary to evaluate how PA might help children with ADHD.

## Methods

### Participants

A convenience sample of 51 guardians (i.e. caregivers and parents) from a large metropolitan city were invited to report on their children’s ADHD, PA and engagement in PA. A majority of guardians were female and 70% of children on which they reported were male (*M* = 0.30, *SD* = 0.46). A majority of guardians identified as Canadian, and most did not specify their ethnic identity. However, the sample represented a middle-to-slightly higher socio-economic bracket as the median reported income was between $100,000 and 149,999. The median income in the jurisdiction of the study is approximately $100,000. Guardians were sought for two reasons. First, collecting data from guardians allowed the authors to employ an online survey format. Second, collecting data from guardians allowed the authors to evaluate the feasibility of the format for future research especially critical now given reductions with in-person access during COVID-19. Participating guardians were required to have: (1) at least one child within the ages of 6–12 years with a primary DSM-IV and/or DSM-5 diagnosis of ADHD, (2) lived with the child for at least the past year, (3) served as legal guardians and (4) fluency in English. Participating guardians were emailed a consent form and password to securely access the following four online questionnaires administered using a password-protected Google Form: (1) the ADHD Rating Scale-IV (ADHD RS-IV; DuPaul et al., 1998), (2) the ADHD Impairment Rating Scale (ADHD IRS; Fabiano et al., 2006), (3) the Physical Activity Questionnaire (PAQ; Aranas, 2021) and a version of (4) the Child and Family Information Questionnaire (CFIQ; Jiang, 2021).

## Materials

1. The ADHD RS-IV (DuPaul et al., 1998) is an established 18-item norm-referenced survey designed to assess a child’s ADHD symptoms within the last 6 months. If children were taking medications for ADHD, guardians were instructed to rate their children’s functioning as best they could when children were off medications to capture the effect of PA on ADHD symptoms without the contributing effects of medication. Children’s medication status was also measured with the CFIQ (see below). Although PA is envisioned to be a complementary treatment to other treatments, initial empirical evaluation of the association between PA and children’s symptoms should be considered in isolation as well.

2. The ADHD IRS (Fabiano et al., 2006) is a well-established 6-question survey designed to assess a child’s ADHD impairment in seven domains, including relationship with peers, siblings and parents, and academic progress, self-esteem, influence on family functioning and overall impairment. For each question, guardians are asked to respond using a scale that indicates whether their child needs treatment and/or special services. Because the response scale involves guardians commenting about level of treatment needed, guardians were not given additional instructions to consider their child’s impairment without medication as we anticipated this would have confused their responding.

3. The PAQ was designed by the first author to help guardians report on children’s PA (Aranas, 2021). The first part of the PAQ prompts guardians to identify and enumerate a child’s PA, if any, occurring more than once in the past 6 months. For each PA, guardians report the intensity, frequency and duration. Reports of intensity were measured with a 4-point Likert-type scale ranging from “*Never sweating and becoming breathless*” to “*Very heavy sweating and becoming breathless*.” Frequency of each PA activity was measured in days per week and months per year, and the duration of each PA activity was measured in hours and minutes per session. In the second part of the PAQ, guardians were asked to report on their prioritization of their child’s engagement in PA (*Priority*), their perception of their child’s enjoyment of PA (*Enjoyment*) and their efforts in engaging their child in PA (*Engagement*) using a 5-point Likert-type scale ranging from “*Extremely*” to “*Not at all*.” No psychometric analyses were conducted on the PAQ because guardians did not complete the same number of ratings due to differences in number of PAs reported. Thus, internal consistency could not be computed due to missing cases, and test–retest was impractical given the cross-sectional nature of the study.

4. The CFIQ is a two-part questionnaire with a total of 38 questions designed to assess the child (Part I) and the guardian’s (Part II) demographic characteristics, mental health issues and treatment or intervention information (Jiang, 2021). Importantly, the CFIQ probes the child’s current treatment and history in order to account for these variables in the analysis.

### Power analysis

A power analysis (G*Power 3.1; Faul et al., 2009) was conducted to evaluate the sample size required for the present study. In order to achieve a minimum power level of .80, a fixed effects model was used with a type-I error set at .05, an estimated PA effect of *f* = 0.35, and three predictors (i.e. intensity, frequency and duration of PA). Given these parameters, a minimum total sample size of 36 was required. Although previous studies have yielded small to medium effects of PA on children with ADHD and autism spectrum disorders (Tan et al., 2016), medium to large effects have also been found (Cerrillo-Urbina et al., 2015). A medium/large effect was assumed to target a realistic sample size for recruitment.

## Results

### Descriptive statistics

All analyses were conducted with IBM SPSS Statistics (Version 27 for Mac). Table 1 shows the descriptive statistics. Cronbach’s alpha for the ADHD RS was .92 and .69 for the ADHD IRS; .70 is typically considered an acceptable level of internal consistency (Nunnally, 1978). As shown in Table 2, the ADHD RS and IRS scales were significantly correlated (*r* = .43, *p* = .002). Also, two dimensions of reported PA levels (i.e. intensity and duration) were significantly correlated (*r* = .60, *p*<.001). Given the high correlation in these two PA dimensions and to avoid multicollinearity in subsequent regression analyses, these dimensions were transformed into Z variables and combined to form an aggregate PA variable (i.e. PA Aggregate). PA Aggregate was significantly associated with ADHD RS (*r* = .29, *p* = .039) but not IRS (*r* = −.01, *p* = .964). All PA engagement questions (i.e. priority, enjoyment and engagement questions) were also highly correlated, so they were transformed into Z variables, and aggregated into a single variable (Engagement Aggregate) to avoid multicollinearity. Engagement Aggregate was significantly correlated with PA Aggregate (*r* = .50, *p*<.001) but not ADHD RS or IRS.

**Table 1.** Descriptive statistics.

Variables	Min	Max	*M*	*SD*
Child age (in years) Child gender (0 = male; 1 = female) Identify as Canadian (0 = Not at all; 10 = Completely) Household income Age at diagnosis Sibling* Currently taking Medications for ADHD* Other treatments* Past comorbidities* Current comorbidities* BPT effectiveness (0 = Very ineffective; 7 = Very effective) ADHD RS ADHD IRS Intensity Frequency Duration Priority Enjoy Engagement	6 0 3 <$5000 3 0 0 0 0 0 2 28 7 0 0 0 6 3 5	12 1 10 >$200000 10 1 1 1 1 1 7 72 28 4 336 600 89 87 82	8.66 0.30 9.40 $100,000-149,999** 6.41 0.88 0.74 0.34 0.64 0.52 4.70 59.02 19.88 2.53 125.57 116.47 31.56 31.56 30.52	1.59 0.46 1.60 1.57 0.33 0.44 0.48 0.49 0.51 1.19 10.10 4.77 1.35 98.76 117.12 19.32 19.14 18.67

Note: 0 = no; 1 = yes; **Median.

**Table 2.** Correlations matrix.

Variables	ADHD RS	ADHD IRS	PA aggregate	Engagement aggregate	Age	Age at diagnosis	Currently taking medications for ADHD	Age symptoms were first noticed	Other treatments
ADHD IRS	.43**								
PA aggregate	.29*	−.01							
Engagement aggregate	.23	−.06	.50**						
Age	−.21	−.29*	.14	−.08					
Age at diagnosis	−.30*	−.22	.12	−.12	.59**				
Currently taking medications for ADHD	.22	.01	.17	.27	.10	−.01			
Age symptoms were first noticed	−.42**	−.18	−.04	−.15	.34*	.36*	−.07		
Other treatments	.24	.40**	.09	.09	−.11	−.20	−.15	−.46**	
Perceived BPT effectiveness	.12	−.29*	.18	.29	.20	−.09	−.12	−.15	.20

Note. Gender, siblings, Currently Taking Medications for ADHD, past comorbidities and current comorbidities were not correlated to the outcome variables of interest – RS and IRS.

** *p* < .01.

* *p* <.05.

### Regression analyses

Two hierarchical linear regressions were conducted based on the statistically significant correlations observed in Table 2. The first regression (Table 3) included ADHD RS as the outcome variable, and *age of diagnosis* and *age when symptoms were first noticed* in the first layer of predictor variables. PA Aggregate was included in the second layer of predictors. The second regression (Table 4) included ADHD IRS as the outcome variable, and *age*, *other treatment* and *perceived Behaviour Parent Training (BPT) effectiveness* in the first layer of predictors. Although PA Aggregate was not significantly correlated with IRS as shown in Table 2, it was nonetheless included in the second layer of predictors to evaluate its effect size given the focus of the study and for future sample size recruitment. No regression analyses were conducted with Engagement Aggregate as a predictor of ADHD symptoms and/or impairment because these variables were not statistically related (see Table 2).

**Table 3.** Model summary of hierarchical regression analysis predicting ADHD symptoms.

	Model 1			Model 2		
Variable	*B*	*SE B*	β	*B*	*SE B*	β
(Constant)	72.36	5.74		74.20	5.52	
Age at diagnosis	−0.90	0.92	−.14	−1.29	0.89	−.20
Age symptoms were first noticed	−2.07	0.81	−.37*	−1.89	0.78	−.33*
PA aggregate				1.76	0.74	.31*
*R* ^*2*^		.44			.53	
*F* for change in *R*^*2*^		5.32**			5.62*	

Note. ** *p* < .01.

* *p* <.05.

**Table 4.** Model summary of hierarchical linear regression predicting ADHD impairment.

	Model 1			Model 2		
Variable	*B*	*SE B*	B	*B*	*SE B*	β
(Constant)	28.76	3.54		29.47	3.67	
Age	−0.46	0.37	−.16	−0.51	0.37	−.18
Other treatments	4.04	1.18	.45**	3.98	1.19	.44**
Perceived BPT effectiveness	−1.30	0.49	−.35*	−1.35	0.50	−.37**
PA aggregate				0.27	0.32	.11
*R* ^*2*^		.57			.57	
*F* for change in *R*^*2*^		6.55**			0.68	

Note. ** *p* < .01.

* *p* <.05.

## Discussion

The current study was designed to examine the relationship between PA levels, PA engagement and children’s ADHD symptoms and/or impairment based on parent reports. Amongst the three PA dimensions evaluated in the study, frequency was the only variable that *did not* approach significance in its association with ADHD symptoms and/or impairment. This result suggests that children’s exertion in PA as measured by intensity and duration matters more than the frequency of their participation in PA. That is, a child who engages in PA frequently but does not move much may not reap the effects of PA as expected. Nonetheless, engaging in PA frequently is the first step to learning to exert intensity and doing so for extended amounts of time.

Our findings are congruent with a meta-analysis of 79 studies conducted by Chang and colleagues (2012) on the effects of acute exercise on cognitive performance. In their meta-analysis, Chang and colleagues found that intensity and duration moderated the relationship between PA and cognitive performance. In another meta-analysis, Tan and colleagues (2016) did not find number of sessions of PA, or frequency of PA, to moderate the effect of PA on cognitive functioning. Thus, consistent with the findings of the current study, the aggregate of intensity and duration appear to be key dimensions in the relationship between PA and child functioning. However, the causal relationship between intensity and duration of PA and children’s ADHD symptoms and/or impairment needs to be explored in future studies. Although true experimental studies may be unfeasible for ethical concerns, quasi-controlled studies could be conducted to evaluate whether PA alleviates ADHD symptoms and/or impairment.

Although a significantly positive association was found between children’s PA and engagement in PA as reported by parents, PA engagement was *not* related to ADHD symptoms or impairment. The lack of correlation could be attributed to how engagement was measured. There is potential measurement error in having parents report on a child’s internal volitional state. Nonetheless, a positive association between PA engagement and PA levels was found, suggesting that parents reported higher levels of engagement in PA when children participated in more intense and longer durations of PA. These findings are congruent with previous findings (Gustafson & Rhodes, 2006; Welk et al., 2003).

In terms of accounting for variance in ADHD symptoms and/or impairment, the first hierarchical regression showed that aggregating PA intensity and duration explained a statistically significant portion of the variance in ADHD symptoms, beyond that explained by typical demographic variables (e.g. age of initial symptoms). As anticipated, the coefficient for age of initial symptoms was negative at −.33 indicating that earlier diagnosis is associated with worse ADHD symptoms. Surprisingly, the coefficient for PA aggregate was positive at +.31. At first glance this finding appears to be the exact opposite of what one would expect to find if PA was indeed helping children. However, this positive relationship could be revelatory of how PA serves as an outlet for children who experience significant hyperactivity; for example, children who experience more hyperactivity may find it necessary to engage in *more PA* to help manage their symptoms. This would account for the *positive association* between PA and ADHD symptoms. It is also possible that the 6-month window used for measuring PA and ADHD symptoms was too narrow to reveal the longer-term association between PA and ADHD symptoms. Although the questionnaire probed parents to report PAs in the last 6 months, guardians did not specify the length of time (e.g. in months or even years) of each PA. A *negative association* between ADHD symptoms and PA could have been missed due to this narrow time span.

The second hierarchical regression showed that PA aggregate *did not* account for a statistically significant portion of the variance in ADHD impairment beyond that accounted by other typical variables (e.g. other treatments and perceived BPT effectiveness). However, the coefficient of .11 was positive as in the first regression. Again, this positive coefficient could be interpreted to suggest that children who experience the most impairment use PA as an outlet. Alternatively, it is possible that our measurement failed to capture the longer-term trend. Notwithstanding, the nonsignificant relationship between PA aggregate and ADHD impairment, a positive coefficient of .44 was found between the use of other treatments and ADHD impairment. This positive association suggests that children with more impairment are involved in more treatments to lower impairments. Perceived BPT effectiveness, however, was associated with lower ADHD impairment as expected. The coefficient of −.37 indicates that when BPT effectiveness is rated as low, ADHD impairment is viewed as higher. Future research needs to include questions about the length of time of all treatments so as to account for the window of time for efforts to reduce ADHD symptoms and impairments.

### Limitations and directions for future research

This study has several limitations. First, the PAQ was newly designed as a means to measure parental reports of PA intensity, frequency, duration and PA engagement in their children. The PAQ was created because to the author’s knowledge, there are no existing parental reports of physical activity questionnaires in the literature that captures different dimensions of PA in children. Although the freely available International Physical Activity Questionnaire (IPAQ, 2016) could have been adapted for this study, this was not done for several reasons: First, the IPAQ would have required surveying children directly; second, it was developed for an older age group (15–69 years); and third, relevant questions involve skip routines that would have required a more sophisticated platform than Google forms for administration. Nonetheless, it may be worthwhile in future studies to consider adapting the IPAQ to directly assess younger children’s self-reports of their PA.

While the PAQ we developed was based on previous measurement of physical activity used in the literature, its validity and reliability has not yet been established for reasons mentioned in the *Method* section. Future work should consider modifying the PAQ (e.g. requiring the same number of ratings from all respondents) so that it can become a measure that permits internal consistency analyses to be done. Moreover, as noted in the previous section, the PAQ should also be revised to include questions about the *specific length of time* for each PA reported. Asking parents to simply list their children’s PAs within the last 6 months lowers the sensitivity of the measurement tool for capturing the most accurate association between an intervention and its effects.

Second, the sample size was relatively small. Thus, the current study could have been under powered, risking the detection of a significant association between PA and ADHD impairment (measured with the IRS in the present study). Larger samples are needed to help clarify the relationship between PA and ADHD symptoms and impairment. Moreover, future studies need to ascertain the ethnic identification of the sample.

Third, the study relied on guardians’ self-reports of their children’s ADHD and PA. Although self-reports, in the form or questionnaires, are the most common and feasible method of measuring PA at the population level (Adamo et al., 2009), the risks involve the over-/under-estimation of information. Reliability may be compromised by memory biases, and validity may be compromised by social-desirability biases (Adamo et al., 2009). Because self-report methods possess several limitations in terms of reliability and validity, future research requires the use of more objective measures in the assessment of PA in conjunction with self-reports. Physiological markers such as heart rate monitoring and respiratory rate, motion sensors such as accelerometers and pedometers, and direct observations may be used as less biased measures (Adamo et al., 2009). Additionally, there is the issue of a *parent by proxy problem* which suggests that parental ratings are not interchangeable with a child’s ratings of activities (Galloway & Newman, 2017). Indeed, it is increasingly necessary to consider a child rights perspective in the way we investigate children’s ADHD functioning and treatment by involving children’s direct voice and participation in assessment research. Although Federal law in the United States supports services and accommodations for students with disabilities such as ADHD, there are other considerations to consider in research such as the level of children’s direct participation to answer questions about their symptoms and impairment in cognitive and social-emotional domains. Toward this end, as mentioned previously, future studies should consider adaptations of the IPAQ so as to measure the perspective of the child directly regarding their symptoms, impairment and PA engagement.

Fourth, while Visser and colleagues (2013) contend that primary caregivers are fairly accurate reporters of ADHD diagnosis, ADHD diagnosis is complex and requires reports about the child’s functioning in different settings (American Psychological Association [APA], 2013). The present study aimed to recruit guardians whose children had a professional diagnosis of ADHD. However, verifying the diagnosis was not possible. Future studies may need to verify ADHD diagnosis by administering rating scales such as Conners-3 (Conners, 2008) or conducting clinical interviews with a qualified clinician. Future studies should also consider using teacher reports to measure ADHD symptoms and impairment in the classroom.

Fifth, given the scope of the original study, children’s social and emotional functioning was not explicitly measured. However, assessing children’s level of social and emotional wellness is necessary for future work. Children who have ADHD are at risk for anxiety and depression, and PA may indeed be associated with alleviating these states. This would add yet another dimension to a rationale for including PA as a supplemental treatment for children with ADHD.

### Implications and conclusion

Figuring out how to properly measure the relationship between PA and ADHD may be the first step towards understanding how PA is associated with ADHD and how guidelines can be accurately developed for children with ADHD. At present, this study highlights the positive association between children’s physical activity and ADHD symptoms. Although the results of this study have provided some answers to the research questions posed, perhaps the overarching conclusion is that we need better measurement tools to evaluate PA in children with ADHD. For example, we have no clarity on what the optimal levels of PA are to potentially maximize the benefits of PA for those children with varying levels of ADHD.

PA continues to be a variable worthy of study. It represents a potentially low-cost alternative but its long-term effects in relation to current evidence-based treatment options are lacking (Rommel et al., 2015). Sanchez and colleagues (2005) contend that very few individuals with ADHD are effectively treated throughout the course of their diagnosis. As such, treatment gains tend to be short-lived, with limited, if any, long-term beneficial effects. Although PA may not ever replace established treatments, it could be used to supplement medication and/or behaviour therapy. Moreover, introducing PA to children with ADHD may help establish a path of healthy behaviours that offers life-long physical and mental health. It is therefore important to consider alternative and adjunct options to provide children with more options for dealing with ADHD than is currently the case. PA may be especially helpful for families who do not have access to medications or behaviour therapy or are looking for more sustainable, low-cost alternatives.
